# Decadal Variations in Eastern Canada’s Taiga Wood Biomass Production Forced by Ocean-Atmosphere Interactions

**DOI:** 10.1038/s41598-017-02580-9

**Published:** 2017-05-26

**Authors:** Etienne Boucher, Antoine Nicault, Dominique Arseneault, Yves Bégin, Mehdi Pasha Karami

**Affiliations:** 1 University du Québec à Montreal, Dépt. of Geography and GEOTOP, Montreal, H2V 1C7 Canada; 2 ECCOREV, FR 3098, CNRS/Aix-Marseille Université, Europôle Méditerranéen de l’Arbois, BP 80, 13545 Aix-en-Provence cedex 4, France; 3 University du Québec à Rimouski, Dept. of Chemistry, Biology and Geography, Centre d’études nordiques, Rimouski, G5L 3A1 Canada; 4 Centre Eau Terre Environnement, Institut National de la Recherche Scientifique, Centre d’études nordiques, 490 de la Couronne, Québec, G1K 9A9 Canada; 5 Dept. of Atmospheric and Oceanic Sciences, McGill University, Montreal, H3A 0G4 Canada; 6 Rossby Centre, Swedish Meteorological and Hydrological Institute, Norrköping, SE-601 76 Sweden

## Abstract

Across Eastern Canada (EC), taiga forests represent an important carbon reservoir, but the extent to which climate variability affects this ecosystem over decades remains uncertain. Here, we analyze an extensive network of black spruce (*Picea mariana* Mill.) ring width and wood density measurements and provide new evidence that wood biomass production is influenced by large-scale, internal ocean-atmosphere processes. We show that while black spruce wood biomass production is primarily governed by growing season temperatures, the Atlantic ocean conveys heat from the subtropics and influences the decadal persistence in taiga forests productivity. Indeed, we argue that 20–30 years periodicities in Sea Surface Temperatures (SSTs) as part of the the Atlantic Multi-decadal Oscillation (AMO) directly influence heat transfers to adjacent lands. Winter atmospheric conditions associated with the North Atlantic Oscillation (NAO) might also impact EC’s taiga forests, albeit indirectly, through its effect on SSTs and sea ice conditions in surrounding seas. Our work emphasizes that taiga forests would benefit from the combined effects of a warmer atmosphere and stronger ocean-to-land heat transfers, whereas a weakening of these transfers could cancel out, for decades or longer, the positive effects of climate change on Eastern Canada’s largest ecosystem.

## Introduction

Each year, nearly 20% of the world’s forest carbon stock is sequestrated by boreal biomes^1^. In Eastern Canada (EC), this stocking primarily results from the growth of a single tree species, black spruce (*Picea mariana* Mill.), which dominates the entire ecosystem from its southern fringe (49 °N) to the tree line (57 °N). Over decadal to inter-decadal time scales, the fate of this biome and its overall efficiency for trapping carbon are thus closely tied to the response of the dominant species to climate variability.

Taiga forests of EC located in the unlogged part of the boreal biome (north of 52 °N) are generally considered low-productivity ecosystems, limited by cold temperatures and short growing seasons^2^. However, ongoing and projected CO_2_-driven warming trends for the next decades might attenuate those limitations by promoting warmer and longer growing seasons^3^. Yet, to what extent those changes could benefit to taiga forests in EC remains unclear due to the scarcity of data-based studies investigating links between black spruce forest growth and climate variability in the area. Another level of uncertainty stems from the fact that naturally occurring climate variability may superimpose, amplify or even counteract the monotonic effects associated with anthropogenic warming on black spruce productivity. For example, if black spruce growth is a thermo-dependent process, then mechanisms that govern heat transport and exchanges at subarctic latitudes need to be investigated to determine how they can persistently alter forest productivity and carbon sequestration capacity.

Both atmospheric and oceanic dynamics should be invoked as potential internal climate variability factors that can drive persistent changes in the productivity of taiga forests in EC. The NAO^4^ represents a dominant winter mode of atmospheric variability. It resumes infra-annual to multi-decadal variations in the strength and direction of storm tracks over the North Atlantic sector^5^. The NAO drives an array of climate variations over North America and Europe^6–8^, with a possible extended effect on forest growth during summer in EC, as evidenced by tree ring studies conducted in the area^9, 10^. Other studies have also emphasized the important influence of North Atlantic Sea Surface Temperatures (SSTs) on the state of the atmosphere during the warm season^11^, especially to address climatic phenomena that persist on timescales longer than decades and for which oceans are most commonly assumed to play a determinant role. Those low-frequency variations are usually summarized by the Atlantic Multi-decadal Oscillation (AMO^12^), a horseshoe pattern that describes SST low-frequency (decadal to multi-decadal) fluctuations in the North Atlantic. Despite of the fact that the physical mechanisms driving the AMO remain unclear (see the recent work by Clement *et al*.^13^), the impact of the AMO on long-term circum-Atlantic temperature and precipitation variability is unequivoqual^14–18^.

**Figure 1:**
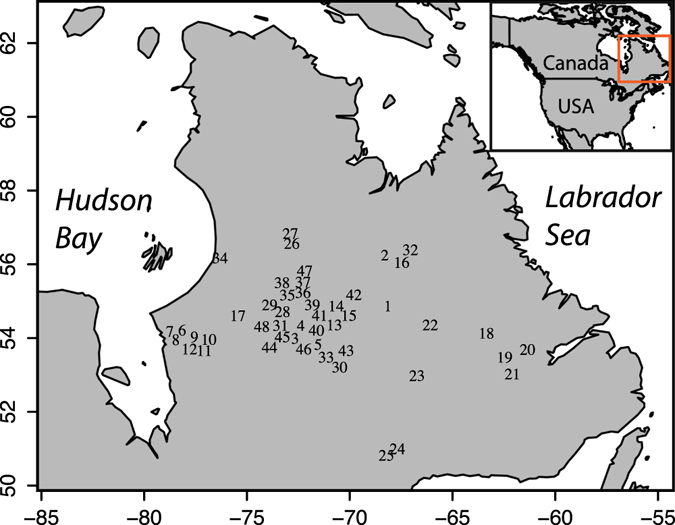
Tree ring sampling sites in Eastern Canada (EC). Map generated in R^63^.

Here, we use a highly replicated network of tree ring measurements from EC’s taiga forests (Fig. 1) to unravel the possible influence of oceanic and atmospheric natural climate variability on wood biomass production. The network fully covers the unmanaged portion of EC’s taiga forests, north of 52 °N. We designed a novel wood mass indicator (*MW*) calculated from both ring width and ring density measurements, and we analyzed correlations with major climatic drivers such as temperatures and precipitation, as well as teleconnections with North Atlantic sea surface temperatures (AMO variability) and atmospheric dynamics (NAO variability). Our study reveals that phases of decadal to inter-decadal, persistently high (or low) forest growth in EC were triggered by low-frequency variations in the strength of heat transfers from the North Atlantic. We conclude that ocean-generated fluctuations in climate can have a significant impact on EC’s forest productivity, possibly obscuring the effects of human-induced temperature changes.

## Results

**Figure 2:**
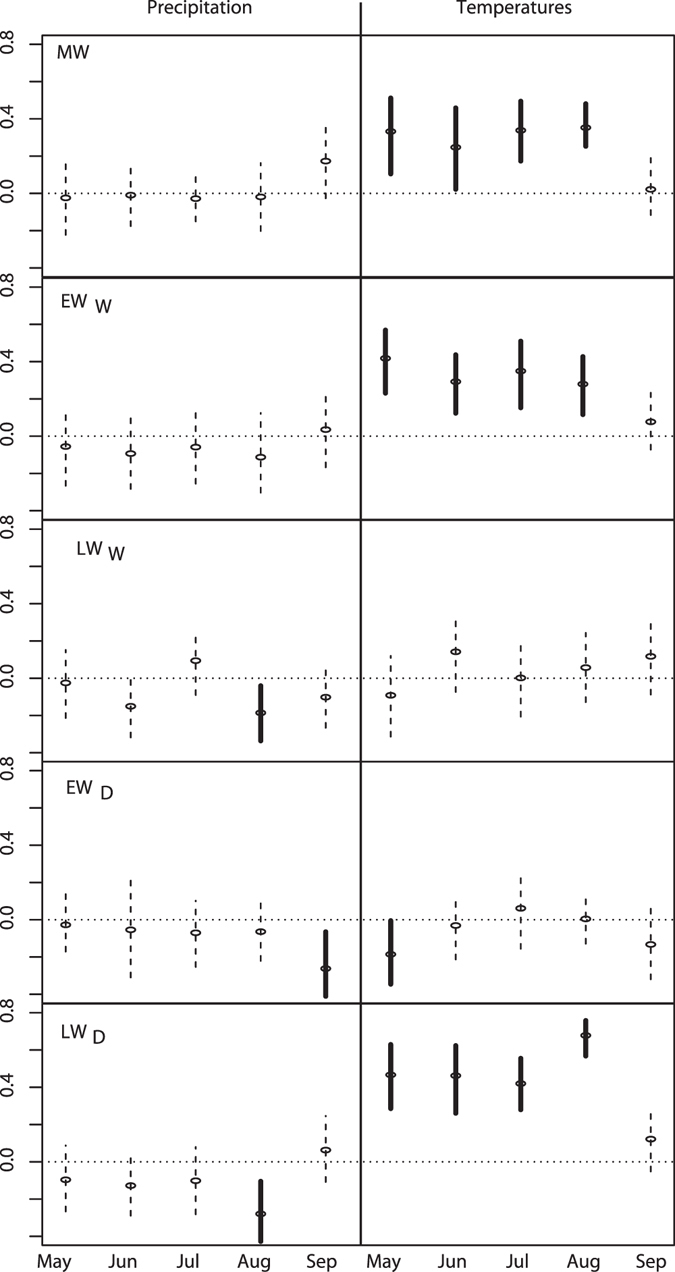
Pearson correlation coefficients for *MW* (top) and the four variables used in the calculation of *MW*: *EW*_*w*_, *LW*_*w*_, *EW*_*D*_ and *LW*_D_. Relationships with precipitation and surface temperatures are shown in the left and right panels, respectively. Bold lines indicate significant (*p* < 0.05) relationships. All correlations are calculated on the 1901–2000 period (N = 100) after averaging both climatic and tree ring data from the 48 sites used in this study.

**Figure 3:**
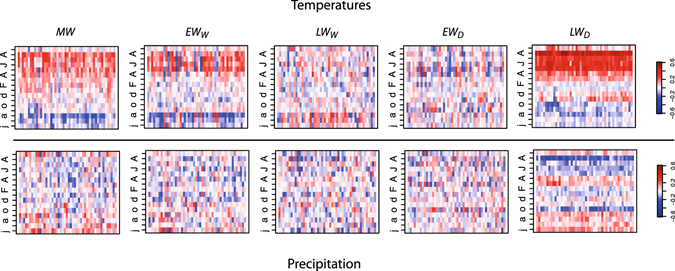
Pearson correlation coefficients for *MW* (top) and the four variables used in the calculation of *MW*: *EW*_W_, *LW*_W_, *EW*_D_ and *LW*_D_ at each site investigated (N = 48, sites on the x-axis, ranked (from left to right) from 1 to 48).

**Figure 4:**
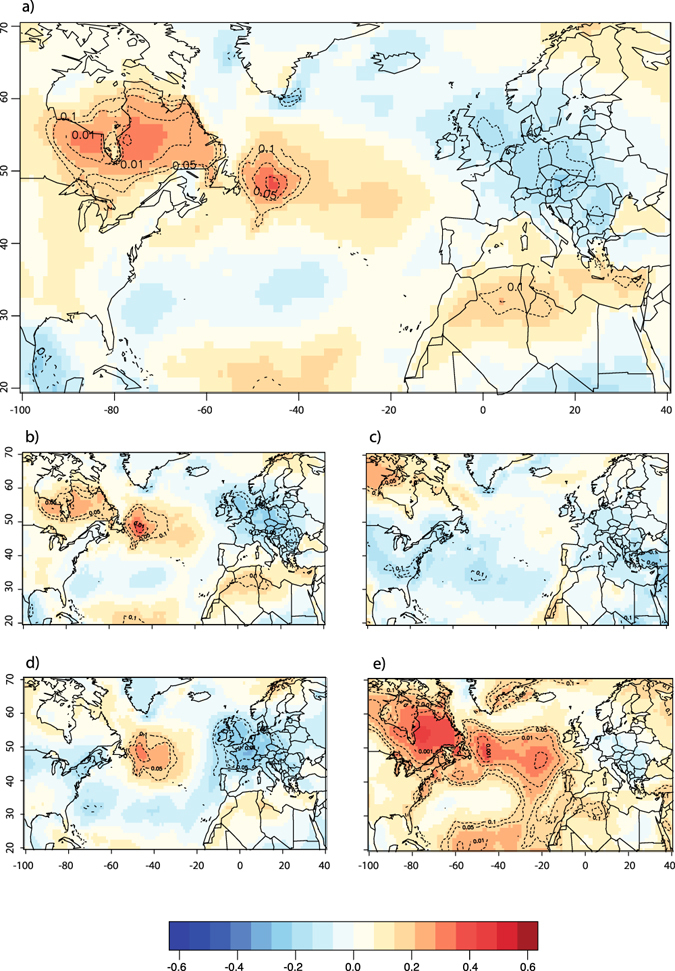
The regional growing season (May-August) surface air temperature signal captured by *MW* (**a**), *EW*_*W*_ (**b**), *LW*_*W*_ (**c**), *EW*_*D*_ (**d**) and *LW*_*D*_ (**e**). Map shows Pearson correlation coefficients between tree ring based index (averaged across all 48 sites in Quebec-Labrador) and surface air temperatures across the North Atlantic basin. Dotted lines represent *p* values for these correlations, after taking into account serial autocorrelation^62^. Maps generated in R^63^.

Wood biomass production (*MW*) in EC’s taiga is primarily controlled by growing season temperatures (Fig. 2). The correlation coefficients between regionally averaged *MW* and May to August temperatures are all positive (all *r* > 0.3, *p* < 0.05), indicating that warm springs/summers positively influence wood biomass production. The spatial coherency of the response to temperatures across the investigated area is another indication of the influence of growing season temperatures on black spruce forest productivity. Indeed, the correlations with May to August temperatures are mostly positive (Fig. 3), irrespective of the site’s position in EC (no clear pattern emerges, however). Consequently, the regionally averaged *MW* is imprinted by a strong and significant regional surface temperature signal. During the growing season, *MW* correlates well with surface temperatures over much of EC, from the western margin of the Hudson Bay to the Labrador Strait (Fig. 4a). Significant correlations with *MW* also extend eastward and reach the western portion of the Atlantic ocean (about 1000 km East of Newfoundland, Canada). Our analysis only reveals a weak influence of precipitation on *MW* both at the site scale and the regional scale.

Not all components used to calculate *MW* respond equally to temperature variability during the growing season (Fig. 4b–e). Earlywood densities (*EW*_*D*_) and latewood widths (*LW*_*W*_) are both very poorly correlated with growing season temperatures or precipitation Fig. 3. Conversely, earlywood widths (*EW*_*W*_) and latewood densities (*LW*_*D*_) both respond similarly to temperatures and precipitation over the 1901–2000 period, although with different strengths. Compared to *MW*, *EW*_*W*_ presents slightly weaker relationships with temperatures and tends to be better associated with spring temperatures (Figs 2, 3 and 4b). However, *LW*_*D*_ appears to be very responsive to growing season temperatures (*r* = 0.69, *p* < 0.01), even more than *MW* or *EW*_*W*_. In fact, *LW*_*D*_ presents the strongest and most spatially coherent response of all proxies forming *MW*. The strength of this response is mostly attributable to a clear and highly significant positive effect of August temperatures on *LW*_*D*_ (Fig. 2), visible across Quebec-Labrador (Fig. 3). Indeed, the site-specific correlation coefficients are all systematically higher than 0.55 (*p* < 0.01) for August temperatures. The average *LW*_*D*_ also carries a strong regional surface temperature signal, even stronger than the one imprinted into the *MW* series (Fig. 4e). The regional surface temperature signal resonates over much of the North Atlantic ocean, where temperatures offshore of Newfoundland appear to be significantly correlated with *LW*_*D*_.

**Figure 5:**
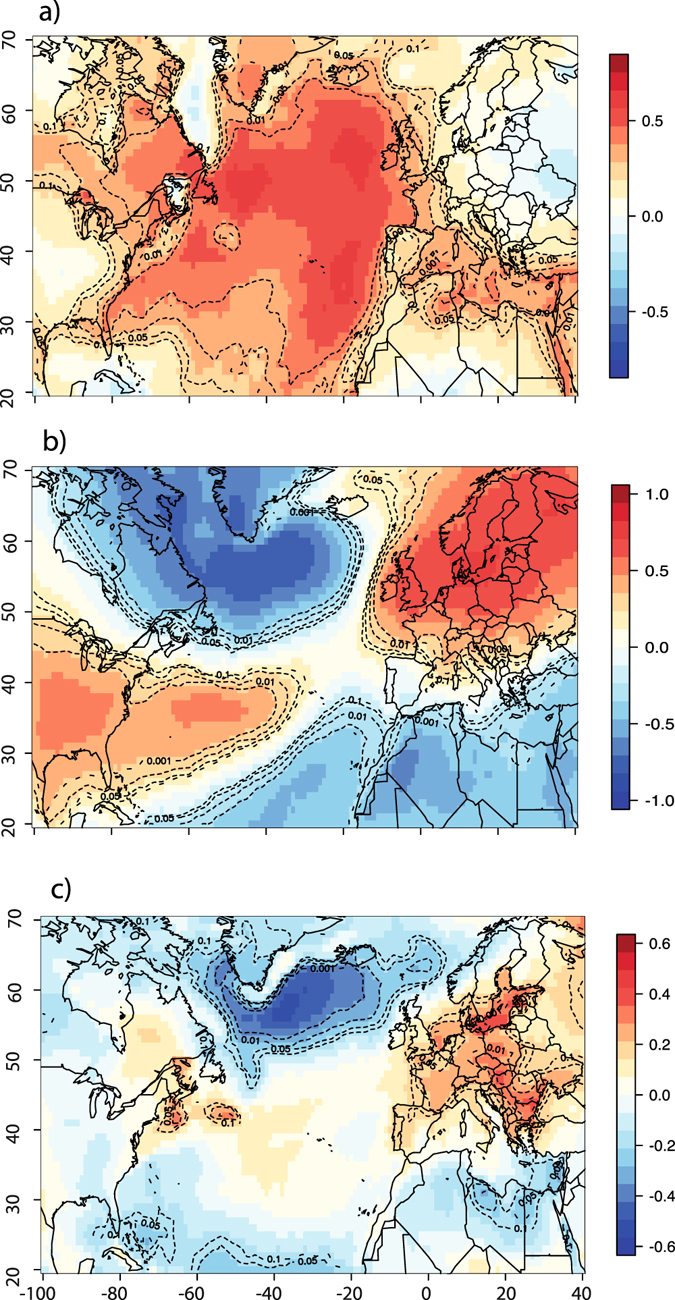
Analysis of ocean-atmosphere teleconnections across the North-Atlantic. Correlations between surface air temperatures and AMO during the growing season (**a**), between NAO and surface air temperatures during the winter season (DJF) (**b**), and between winter NAO (DJF) and surface air temperatures during the growing season (**c**). Dotted lines represent *p* values for these correlations, after taking into account serial autocorrelation. Map generated in R^63^.

Growing season temperatures that control *MW* and its components across EC are positively associated with variations in SST across the Atlantic. The summer AMO_*May*–*Aug*_ index that summarizes variations in Atlantic SSTs during the warm season correlates well with the growing season temperatures over EC (*r* = 0.5, *p* < 0.05) during the twentieth century (Fig. 5a). In fact, in the circum-Atlantic area, EC represents the sector where growing season surface temperatures are most strongly related to SST variability, as described by the summer AMO index. Regions of EC correlated with summer AMO variability include both sides of the Hudson Bay and extend eastward to the coast of the Labrador Sea, where maximal associations between surface temperatures and summer AMO are found. No correlation could be found between AMO_*May*–*Aug*_ and growing season precipitation variability in the study area. Conversely, the NAO pattern has a strong influence on winter (Dec-Feb) temperatures in the circum-Atlantic area, generating colder conditions in EC during periods of positive winter NAO (Fig. 5b). However, the winter NAO does not appear to be significantly related to growing season temperatures in EC (Fig. 5c).

**Figure 6:**
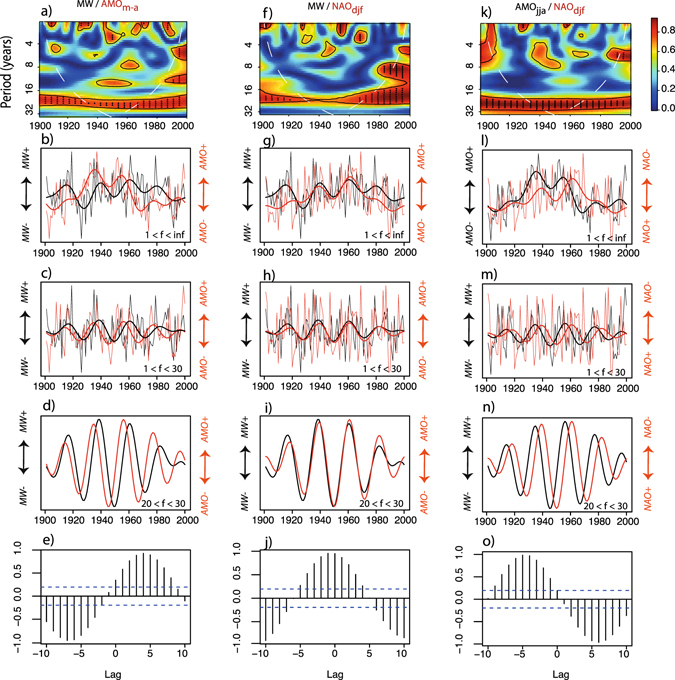
Relationships between MW and the summer AMO (first column), between MW and winter NAO index (second column). (**a**) Cross-spectral coherency analysis between MW and summer AMO, with significant coherencies appearing in bold. (**b**) Raw (full spectrum) MW and summer AMO time series. (**c**) Both time series with frequencies (*f*) > 30 years filtered out. (**d**) Both time series with frequencies <20 years and >30 years filtered out. (**e**) Lagged correlation analysis performed on 20 < *f* < 30 series (**d**), lagging AMO. The second and third columns are identical to the first one; however, the series compared are MW and winter NAO (second column, (**f**–**j**) and winter NAO and summer AMO (third column, (**k**–**o**).

Because EC’s surface temperatures are highly responsive to the AMO, biomass production in this region is also affected by SST variability. Indeed the correlation between summer AMO and *MW* is 0.34 (*p* < 0.05) over the last century. Interestingly, a cross-spectral coherency analysis also highlights a fairly high (>0.7) and significant (<0.1) coherency for periodicities between 20 and 30 years during the same period (Fig. 6a). Thus, our analysis suggests that *MW* and AMO_*May*–*Aug*_ might share a comparable decadal variability component, despite the fact that the coherency at the inter-annual scale remains modest. The coherency between spectra is even more visible when lower frequencies (*f* > 30 years) are filtered out from both *MW* and the AMO series (Fig. 6c). Ultimately, isolating frequency bands comprised between 20 and 30 years (Fig. 6d) allows magnification of the spectral coherency. Moreover, a lagged correlation analysis reveals that filtered (20–30 years) variations in summer AMO systematically precede those of *MW* by approximately 4–5 years within the same frequency band (Fig. 6e). Interestingly, winter NAO also shares coherent 20–30 year periodicities with both *MW* (Fig. 6f–j) and summer AMO series (Fig. 6k–o) and could be identified as a potential source of decadal variability and persistence in black spruce growth at the centennial scale.

**Figure 7:**
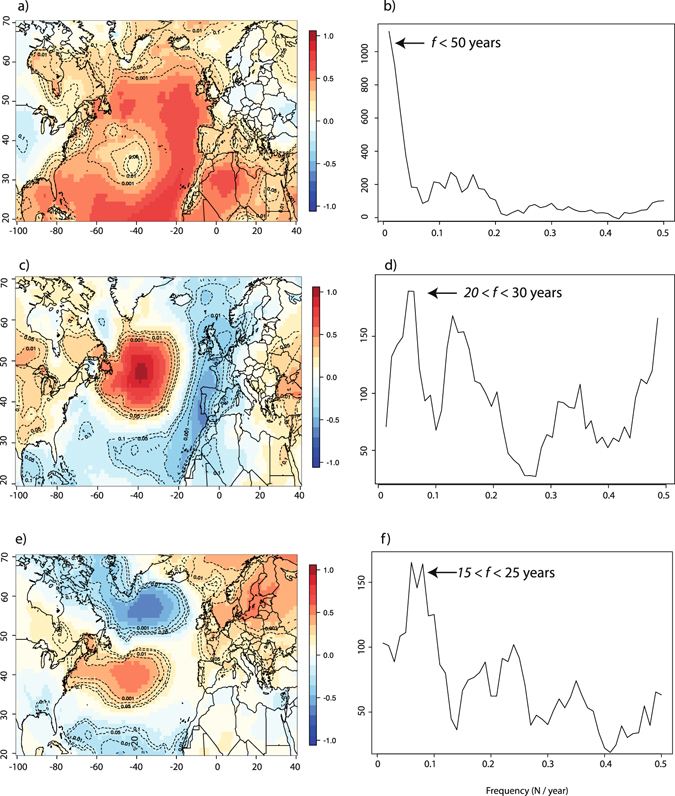
Correlations between the three first PCs of North Atlantic summer SSTs and surface air temperatures: PC1 (**a**), PC2 (**c**) and PC3 (**e**). The associated spectral power for each PC: PC1 (**b)**, 31% explained), PC2 (**d)**, 11% explained) and PC3 (**f**), 9% explained). Dotted lines represent *p* values for these correlations, after taking into account serial autocorrelation. Maps generated in R^63^.

Ocean-atmosphere interactions that occur in the North Atlantic region significantly affect wood biomass production over decadal to inter-decadal time scales, resulting in spatially coherent responses of black spruce forests to temperatures across EC. The 20–30 years periodicities common to *MW*, summer AMO and winter NAO time series suggest that significant amounts of heat that contribute to the growth of EC’s taiga forests could originate from the North Atlantic Ocean. However, numerous processes evolving at different spatial and temporal scales may control heat transfers towards EC, all of which may be imprinted in the AMO series. In order to highlight these processes, we determined the three main modes of SST variability in the North Atlantic through a principal component (PC) analysis (Fig. 7) and related them to AMO/NAO variability. Indeed, the first PC (PC1, correlation with AMO = 0.65, *p* < 0.01) represents 31% of the overall North-Atlantic SST variability and clearly summarizes the dominant, basin-wide, low-frequency summer AMO variability (Fig. 7a). Fluctuations in PC1 result in generalized warmer surface air temperatures across the subpolar North-Atlantic (Fig. 7a) with warm anomalies forming a horseshoe pattern, reaching northern Europe and then extending westward to the coast of Labrador. PC1 presents a dominant low frequency component with most spectral power around 50 to 70 years (Fig. 7b). The pattern of correlation of SSTs with PC1 is quite similar to the one found between surface air temperature and AMO (Fig. 5a). The second component (PC2, 11% of SST variability, correlation with AMO = 0.42, *p* < 0.01), represents decadal to inter-decadal fluctuations of SSTs over the subpolar region of the North Atlantic ocean. The second PC highlights a particularly strong response of surface temperatures over the western portion of the North-Atlantic basin, south of Greenland. The imprint of PC2 on surface air temperatures across the North-Atlantic basin extends westwards to reach the Quebec-Labrador peninsula in EC (Fig. 7c). Interestingly, PC2 has most of its spectral power between 20 and 30 years (Fig. 7d). Lastly, PC3 (9% of SST variability, correlation with AMO = 0.02, *p* > 0.05) also shows considerable spectral power between 15 and 25 years and reveals a pattern of correlation (Fig. 7e) that closely resembles the one found for NAO and surface air temperatures (Fig. 5b,c).

## Discussion

This study highlights the critical importance of growing season temperatures in determining the pace at which black spruce boreal forests produce and store wood biomass in tree trunks. The evidence for a widespread and spatially coherent dependence of wood biomass production on summer temperatures is based on the investigation of EC’s most extensive tree ring multi-proxy network ever assembled in the unmanaged boreal forest of Quebec-Labrador. The sensitivity of high-latitude species to growing season temperatures is a well-known feature of dendroclimatological studies. For this reason, boreal tree ring series have commonly been used as proxies for past growing season temperatures both in the Canadian^19–21^ and Eurasian context^22–25^. In the study area, growing seasons are most often short, starting in May and ending in late August-early September^26^. Warmer conditions, particularly during growth initiation and growth completion, increase the photosynthetic efficiency and thus synthesis/allocation of carbonaceous materials to tree trunks^27, 28^.

Previous studies that investigated the response of black spruce forests relied strictly on ring width chronologies^29–31^. Based on those measurements, important heterogeneities were found in the relationship between tree ring series and climate across the full boreal biome^32^. In the taiga forest of EC, significant and positive dependence on summer temperatures was restricted to the northernmost populations (>54 °N)^29^. South of 52 °N, temperature have had a negative impact on growth and induced widespread growth declines driven by an increase in both evaporative demand and autotrophic respiration^31^. Further south, in the closed-crown boreal forest, black spruce forest stands shift from precipitation-dependant to thermo-dependant populations around the 49^*th*^ parallel^30^. Our analysis reveals a considerably more coherent response to temperatures across the entire taiga because *MW* is capable of tracking temperature controls throughout the growing season. Indeed, the proxy used to calculate wood biomass production (*MW*) integrates variations in ring width and wood density, during both the earlywood and latewood growth phases. For example, our results revealed that two proxies involved in the calculation of *MW* are strongly thermo-dependent: earlywood widths (*EW*_*W*_) and latewood densities (*LW*_*D*_). The thermo-dependence of *EW*_*W*_ implies that early spring temperatures play a crucial role in the production of wood biomass. Indeed, a detailed investigation of black spruce wood micro-cores revealed that warmer (earlier) springs favor an enhanced growth^33^, mainly because growth initiation occurs sooner and earlywood xylem cells grow faster under such conditions. The response of *MW* to temperatures also appears to be very coherent because *LW*_*D*_ reflects the strong influence of late summer conditions on latewood formation. Relationships between *LW*_*D*_ and late summer temperatures are well established in the literature: thick-walled latewood xylem cells form during warm (and perhaps also dry) summers^34^, therefore augmenting wood biomass produced per unit surface (volume). *LW*_*D*_ has a notable influence on the strength and coherency of the response of *MW* to summer temperatures, but the overall effect on wood biomass is dampened by *LW*_*W*_ (eqs 5 and 6), a very complacent proxy that represents less than a third of the full ring width (31%).

Our study revealed that EC’s growing season temperatures are among the most responsive to AMO variability across the circum-Atlantic area (Fig. 5a), especially at decadal to inter-decadal time scales. The spatial correlation with both growing season temperatures and *MW* are unaltered when periodicities longer than 30 years are filtered out from the summer AMO series (not shown), confirming the relevance of decadal to inter-decadal timescales for the study of processes controlling heat fluxes in the western sub-polar North Atlantic Ocean. Based on previous works^35, 36^, we hypothesize that climatic and ecological impacts triggered by summer AMO variability reflect the interactions between two dominant modes of summer SST variability and their respective impacts on surface temperature patterns across the Atlantic basin. For example, from an analysis of observational records (both marine and continental) and GCM modeling results, Frankcombe *et al*.^36^ have highlighted decadal to inter-decadal (20–30 years long) fluctuations in SSTs and subsurface temperatures at subtropical/subpolar latitudes in the North-West Atlantic Ocean. These fluctuations could correspond to those exemplified by our PC2. In fact, 20–30 years fluctuations in summer SST patterns have been attributed to the westward (zonal) migration of surface and subsurface temperature anomalies. The mechanisms invoked to explain the westward migration of temperature anomalies across the Atlantic basin involve the development of thermal Rossby waves forced by antecedent temperature and salinity gradients. Such processes have been theorized in ocean-only models^37^ and represent an important component of SST variability^38^. Such 20–30 year periodicities are clearly discernible in SST and subsurface temperature series^35^, GCM simulations^39–41^ and proxy-based reconstructions of the strength of the Atlantic Meriodional Overturing Circulation (AMOC)^42, 43^ which has commonly been invoked as an important driving force affecting the AMO^42, 44, 45^. Overimposed on decadal to inter-decadal SST variability, Frankcombe *et al*.^36^ have highlighted an additional source of multi-decadal variability in the North Atlantic visible on frequency bands lower than 50 years and most typically associated with the dominant pattern of the AMO. It also presents a characteristic horseshoe pattern much similar to the pattern found by PC1 in the present study. The physical mechanisms causing these basin-wide, low-frequency SST variations in the North Atlantic however remain debated^13^. Frankcombe *et al*.^36^ argued that they most likely result from the modulation of the decadal component of SST variability by meridional temperature and salinity exchanges between North-Atlantic and Arctic waters, further affecting ocean circulation at the scale of the basin. However, from an analysis of slab-ocean models, Clement *et al*.^13^ found that those variations can be triggered by atmospheric circulation only, without any intervention from ocean circulation.

Here, we argue that the North-Atlantic ocean can store the heat affecting decadal to inter-decadal changes in surface air temperatures of surrounding continental masses. However, other processes need to be invoked to explain the redistribution of heat over EC. Interactions with sea ice cover are particularly relevant in that context. This is particularly true for EC, which is surrounded by the Hudson Bay and Labrador Sea, two oceanic systems that undergo significant seasonal freezing during winter. At the scale of the circum-Atlantic region, warmer SSTs contribute to reducing sea ice concentrations in both the Labrador Sea and Hudson Bay during winter. The link between North Atlantic SSTs and sea ice concentrations is supported by state-of-the-art 1000 year modeling experiments, which confirm the negative impact of summer SSTs on the ice cover of these subpolar seas^41^. Sea ice cover has a well-known impact on temperatures at subpolar latitudes, but the effect appears to be strongest in late fall and winter^46, 47^. However, feedbacks from ice cover on atmospheric conditions may also extend up to the warm season. Indeed, recent research has demonstrated that decadal to inter-decadal variations in sea ice concentrations covary with both temperatures and growth of black spruce forests in EC, south of 52 °N^31^.

Whereas the North Atlantic stores the heat required to stimulate EC’s taiga productivity, NAO-driven winter climate variability could contribute to initiate, amplify or increase the persistency of heat transfers from oceans to adjacent lands. Our study has shown that, just like North Atlantic SSTs, the NAO shares coherent decadal to inter-decadal periodicities with both *MW* (Fig. 6f–j) and summer AMO variability (Fig. 6k–o). More precisely, surface air temperature response to PC3 across the Atlantic basin suggests that the winter NAO imprints surface air temperature patterns across the North Atlantic. Surprisingly, common periodicities between *MW*, summer AMO and winter NAO exist despite the fact that NAO variability remains mostly a winter phenomena, with virtually no impact on summer temperatures (Fig. 5c). Hence, NAO-driven climate variability cannot be invoked as a potential factor that directly stimulates wood biomass production in the study area. Nevertheless, recent research has shown that NAO and AMO-type variability interact on decadal to inter-decadal time scales, although some debate remains as to whether the former is a cause or a consequence of the latter^48^. NAO variability can “excite” the ocean oscillator, drive and amplify changes in the vigor of the AMOC^39^ which in turn influences AMO-type patterns of SST variability in the North Atlantic. For example, positive NAO conditions resulting from large surface pressure differences between Iceland and the Azores lead to the strengthening of westerly winds. Stronger westerlies increase surface turbulent heat fluxes over the Labrador Sea and the subpolar gyre. This results in an intensification of deep water formation in the Labrador Sea^40^, in the stimulation of the oceanic circulation and in significant sea ice loss in subpolar Atlantic. Contrastingly, it is well known that the noisier atmosphere has a limited intrinsic capacity to generate, by itself, decadal climatic persistence. Thus, the NAO might, in turn, be imprinted by changes in SST patterns resulting from positive ocean-atmosphere feedbacks^48^. Indeed, warming SSTs during positive summer AMO tend to favor the development of NAO-like pressure gradients over the North Atlantic. The development of such pressure gradients contributes to maintain and intensify the NAO over decadal to inter-decadal time scales. Here, we put forward the idea that winter-based NAO atmospheric variability might modulate SST patterns in the North Atlantic sector, but ultimately, the timescale at which heat is transferred to EC is more likely to be set by the speed at which SST anomalies travel westward, as part of the AMO variability. Further investigation of this ocean-atmosphere feedback mechanism needs to be undertaken by means of coupled GCMs modeling experiments to fully explore its causes and possible consequences on EC’s climate and ecosystems. A promising framework (the delayed oscillator model) has recently been proposed by Sun *et al*.^49^ and could be used to investigate these complex ocean-atmosphere interactions and their impacts on climate variability at the circum-Atlantic scale.

One question remains to be answered: why is the response of black spruce forests to North Atlantic to SST variability lagged by 4–5 years? The delay could have a biological origin and result from a preferential allocation and re-utilization of photosynthetic products stored by trees during years of favorable growth. Even if we took good care of removing the autocorrelation component in our series, it is difficult, for now, to completely rule out this effect without a deeper investigation of high-resolution dendroecological data and associated ecophysiological modeling. Another, perhaps more realistic explanation for this delay would be that ocean-atmosphere interactions that take place in the North-Atlantic sector may take up a few years to amplify to a level that significantly impacts wood biomass production across EC’s taiga. From an ocean-only perspective, several works have confirmed that AMO variability leads changes in air temperature by a few years (3 to 5 years in average) and have associated this characteristic feature to the propagation of a thermal Rossby mode across the sub-polar North-Atlantic^37, 50, 51^. Delays could also be expected with respect to interactions between NAO, AMO and ice cover dynamics at decadal to inter-decadal timescales. For example sea ice anomalies can imprint summer North Atlantic SSTs in a way that increases the persistence of severe sea ice conditions during following years^52^, therefore slowing down the pace at which North-Atlantic SST anomalies may propagate to adjacent lands.

Our findings clarify the response of EC’s largest ecosystem to long-term temperature variability. Based on our analysis, warming temperatures anticipated for the 21st century^53^ are expected benefit growth of EC’s taiga forests. However, increasing temperatures will have a positive effect only up to a certain limit. In the southernmost part of the boreal forest of eastern Canada, excess heat was associated with greater temperature-induced water stress, resulting in large-scale growth declines^31^. In the present study, no such growth decline was observed in the *MW* series, probably because average temperatures are considerably colder in the taiga ecosystem than in the southern boreal region, making temperature-induced growth declines less likely in the unlogged part of the boreal forest. Our study also implied that thermo-dependent biological processes controlling black spruce growth are linked to the state of the ocean and heat transfers from lower latitudes to subarctic environments. Thus, a possible lack of phase between anthropogenic warming trends and AMO-like patterns of SST variability in the North Atlantic could attenuate the positive effects of climate change on EC’s taiga forests. Indeed, proxy-based reconstructions and state-of-the-art modeling studies have pointed to a general slowdown of the AMOC since the beginning of the 20^*th*^ century, - a slowdown that should continue at least during the first part of the 21^*st*^ century^43^. Hypothesizing that AMOC controls part of the AMO (this common belief has however been questioned recently^13^), thus, a slowdown of the overturning circulation associated with colder conditions in the North-West Atlantic could cancel out, for decades or longer, the beneficial effects of human-induced warming on EC’s largest ecosystem.

## Methods

### Tree Ring Series

The tree ring network used in this study is composed of 48 sites spread out across the taiga forest of EC, north of 52 °N (Fig. 1). At each site, stems were selected using strict criteria of tree age, topographic homogeneity and structure. Only dominant trees with a symmetrical shape and free from major anomalies were selected. Collected cross-sections were dried and finely sanded. Tree rings were cross-dated and then measured along two or three radii using a micrometer with an accuracy of 0.001 mm (Velmex Inc., Bloomfield, NY) under a binocular magnifying glass. The accurate dating of each ring was then verified with the program COFECHA^54^. Density measurements were performed at each site following established procedures^55^. Only discs without any anomalies (reaction wood, branches, rotten wood, and so forth) were selected. Three wood samples per tree were precisely cut in 1 mm probes and then placed in a Soxhlet apparatus with ethanol for resin extraction. After a certain acclimation time to ensure constant size and hygroscopic conditions, the probes were X-rayed. To separate latewood from earlywood measurements, the X-ray micrograph was analyzed on a DENDRO 2003 microdensitometer (Walesch, Switzerland). A cellulose acetate calibration wedge^56^ was used to convert the lightness of measurement into density (g/cm3) values. This densitometry analysis provided density and width measurements for both earlywood (*EW*_*D*_ and *EW*_*W*_) and latewood (*LW*_*D*_ and *LW*_*W*_). Cross-dating statistics are presented in Table 1 for each measured proxy.

**Table 1:** Dendroecological information and statistics describing the tree ring network: number of cores and number of trees sampled at each site, Expressed Population signal (eps), Signal-to-noise ratio (snr) and mean correlation between one tree and all remaining trees (rbart. wt).

Site_no	Name	n.cores	n.trees	MW			EW_D_			LW_D_			EW_D_			LW_D_		
				rbar. wt	eps	snr	rbar. wt	eps	snr	rbar. wt	eps	snr	rbar. wt	eps	snr	rbar. wt	eps	snr
1	CANE	16	10	0.57	0.88	4.59	0.53	0.84	5.05	0.27	0.61	1.59	0.17	0.75	3.01	0.46	0.86	6.28
2	CEA	12	8	0.80	0.91	7.62	0.73	0.87	6.78	0.43	0.75	2.97	0.25	0.70	2.30	0.37	0.76	3.18
3	DA1M	18	10	0.72	0.97	18.66	0.67	0.95	17.12	0.50	0.88	7.04	0.28	0.78	3.46	0.42	0.89	7.74
4	DA1X	19	10	0.46	0.82	2.78	0.40	0.77	3.35	0.31	0.70	2.28	0.46	0.79	3.76	0.43	0.87	6.64
5	ESK	9	7	0.59	0.95	9.66	0.57	0.90	8.58	0.50	0.76	3.13	0.60	0.75	3.03	0.47	0.77	3.33
6	GRAFFE	31	23	0.74	0.99	22.94	0.68	0.96	23.73	0.68	0.96	21.81	0.75	0.96	21.11	0.75	0.95	19.33
7	GRAFLG1B	17	14	0.95	0.98	44.83	0.89	0.98	43.26	0.77	0.96	25.49	0.53	0.78	3.63	0.49	0.87	6.50
8	GRAFLG2	22	17	0.87	0.97	13.88	0.81	0.92	11.64	0.72	0.90	9.35	0.12	0.79	3.78	0.25	0.87	6.57
9	GRAFLG3	9	8	−0.08	0.65	3.56	−0.13	0.60	1.50	0.05	0.71	2.46	0.38	0.82	4.43	0.42	0.82	4.65
10	GRAFLG4	10	9	0.78	0.88	6.43	0.73	0.83	4.97	0.67	0.82	4.62	0.82	0.82	4.59	0.76	0.82	4.67
11	GRAFUKB	12	11	0.19	0.74	0.84	0.16	0.67	2.05	0.29	0.83	4.80	0.40	0.60	1.52	0.24	0.61	1.57
12	GRAFVEN	29	20	0.80	0.96	21.16	0.75	0.96	21.04	0.67	0.96	22.24	0.57	0.91	10.61	0.57	0.92	11.55
13	HH1	33	22	0.48	0.94	9.62	0.44	0.89	8.08	0.35	0.89	8.32	0.26	0.87	6.86	0.30	0.92	11.74
14	HM1	39	21	0.65	0.97	30.28	0.59	0.97	29.67	0.26	0.86	6.30	0.36	0.82	4.64	0.37	0.94	16.86
15	HM2	27	23	0.62	0.98	16.39	0.56	0.94	16.92	0.29	0.87	6.55	0.34	0.80	4.06	0.43	0.94	15.04
16	HS1	54	28	0.81	0.97	57.74	0.77	0.98	57.25	0.72	0.99	65.32	0.38	0.88	7.21	0.55	0.96	24.98
17	HU	11	6	0.90	0.90	6.83	0.84	0.88	7.03	0.66	0.77	3.35	0.51	0.67	2.07	0.60	0.87	6.95
18	KAND	9	6	0.58	0.91	7.56	0.54	0.84	5.04	0.24	0.69	2.22	0.68	0.58	1.38	0.50	0.77	3.43
19	LAB17	13	7	0.68	0.79	2.76	0.63	0.72	2.54	0.49	0.64	1.80	0.28	0.68	2.10	0.40	0.86	6.05
20	LAB19	13	7	0.84	0.85	4.68	0.79	0.80	4.06	0.54	0.70	2.35	0.18	0.53	1.11	0.46	0.73	2.76
21	LAB32	16	9	0.76	0.86	3.81	0.71	0.81	4.19	0.51	0.76	3.08	0.32	0.70	2.28	0.28	0.79	3.66
22	LAB35	11	8	0.85	0.81	3.20	0.78	0.76	3.14	0.61	0.53	1.14	0.47	0.61	1.57	0.41	0.70	2.37
23	LAB42	13	8	0.83	0.95	13.62	0.78	0.93	12.56	0.34	0.83	4.89	0.25	0.55	1.23	0.28	0.69	2.20
24	LAB50	16	9	0.60	0.79	5.11	0.57	0.74	2.78	0.35	0.64	1.75	0.27	0.72	2.52	0.45	0.85	5.81
25	LAB56	11	7	0.46	0.91	6.75	0.43	0.86	6.14	0.44	0.73	2.64	0.17	0.56	1.25	0.36	0.80	3.94
26	LAB65	14	7	0.75	0.68	2.61	0.70	0.62	1.59	0.57	0.64	1.76	0.43	0.38	0.62	0.43	0.80	4.03
27	LECA	16	8	0.48	0.96	11.59	0.43	0.91	9.56	0.36	0.86	6.38	0.23	0.70	2.31	0.47	0.90	9.14
28	LECB	15	8	0.81	0.91	17.56	0.76	0.94	16.86	0.70	0.92	12.01	0.31	0.55	1.20	0.39	0.79	3.69
29	LJ1	10	5	0.94	0.93	7.72	0.88	0.88	7.08	0.78	0.88	7.62	0.54	0.75	3.00	0.60	0.85	5.65
30	LJ2	13	7	0.73	0.99	15.50	0.69	0.94	14.51	0.47	0.89	7.90	0.46	0.80	4.11	0.58	0.89	8.29
31	NIT	18	9	0.57	0.88	6.32	0.53	0.84	5.38	0.47	0.80	3.92	0.35	0.77	3.39	0.59	0.91	10.02
32	NLG4	10	5	0.65	0.92	8.51	0.59	0.88	7.38	0.31	0.77	3.27	0.35	0.64	1.80	0.55	0.83	5.00
33	POOL	8	5	0.86	0.85	5.00	0.81	0.79	3.83	0.35	0.38	0.62	0.29	0.61	1.56	0.64	0.85	5.44
34	RM	11	7	0.79	0.88	7.07	0.75	0.82	4.58	0.41	0.68	2.09	0.48	0.75	2.92	0.35	0.77	3.36
35	ROZH	8	4	0.66	0.71	1.71	0.60	0.66	1.90	0.39	0.61	1.57	0.32	0.69	2.20	0.39	0.69	2.18
36	ROZI	10	6	0.77	0.71	2.71	0.70	0.65	1.82	0.47	0.55	1.21	0.42	0.58	1.40	0.60	0.82	4.41
37	ROZM	17	10	0.44	0.92	7.71	0.38	0.87	6.44	0.10	0.77	3.43	0.52	0.68	2.09	0.40	0.80	4.02
38	ROZW	11	7	0.70	0.88	6.51	0.64	0.82	4.45	0.30	0.74	2.83	0.56	0.57	1.30	0.58	0.84	5.08
39	ROZX	12	7	0.38	0.82	4.72	0.34	0.79	3.72	0.32	0.72	2.62	0.18	0.57	1.34	0.32	0.77	3.30
40	RT426	10	6	0.67	0.89	7.29	0.63	0.85	5.62	0.50	0.71	2.39	0.12	0.64	1.77	0.47	0.86	5.89
41	RT485	11	6	0.77	0.79	4.72	0.74	0.74	2.83	0.56	0.59	1.46	0.44	0.60	1.52	0.59	0.78	3.45
42	RT630	7	5	0.91	0.89	7.45	0.88	0.84	5.28	0.35	0.51	1.06	0.58	0.61	1.56	0.55	0.73	2.75
43	RX	12	6	0.78	0.97	24.94	0.73	0.96	21.89	0.65	0.93	13.25	0.33	0.72	2.60	0.53	0.67	2.04
44	T1	20	11	0.40	0.79	3.86	0.36	0.77	3.31	0.24	0.63	1.71	0.45	0.85	5.71	0.49	0.87	6.80
45	T4S	11	6	0.42	0.66	0.83	0.38	0.62	1.65	0.28	0.61	1.55	0.36	0.75	2.99	0.36	0.79	3.65
46	THH	23	13	0.49	0.88	3.84	0.46	0.83	4.82	0.26	0.73	2.69	0.46	0.85	5.67	0.55	0.90	9.39
47	TOM	25	22	0.47	0.86	5.53	0.43	0.82	4.45	0.38	0.79	3.75	−0.12	0.56	1.25	0.11	0.77	3.38
48	TS	18	10	0.81	0.97	11.93	0.77	0.92	10.91	0.43	0.82	4.60	0.45	0.87	6.46	0.50	0.89	8.00
AVERAGE		16.25	10.17	0.66	0.88	10.44	0.61	0.83	9.55	0.44	0.76	6.40	0.38	0.71	3.38	0.46	0.82	6.39

### Wood Biomass Index (*MW*)

In the present study, tree ring width and density measurements (for both latewood and earlywood) were used to calculate the annual biomass of wood produced by black spruce forests at each site investigated. Basal area increments (BAIs) were calculated from tree ring widths as follows (assuming a circular cross-section):

1 $$BA{I}_{t}=\pi {R}_{t}^{2}-\pi {R}_{t-1}^{2}$$

where *R*_*t*_ and *R*_*t*−1_ are the stem radial increments at the end and beginning of a given annual ring, respectively. For thin slices of wood, it is straightforward to show that BAI (cm^2^) is directly proportional to basal volume increment (BVI) (cm^3^). Therefore:

2 $$BV{I}_{t}\equiv BA{I}_{t}$$

BVI measurements can therefore be decomposed in earlywood and latewood fractions:

3 $$BV{I}_{total(t)}=BV{I}_{EW(t)}+BV{I}_{LW(t)}$$

Proportions of BVI formed in each fraction can be estimated from *EW*_*W*_ and *LW*_*W*_ as follows:

4 $$BV{I}_{EW(t)}=BV{I}_{total}\times E{W}_{W(t)}/(E{W}_{W(t)}+L{W}_{W(t)})$$

5 $$BV{I}_{LW(t)}=BV{I}_{total}\times L{W}_{W(t)}/(E{W}_{W(t)}+L{W}_{W(t)})$$

One can finally convert the respective ring volumes (cm^3^) into mass units (g) using density measurements (D, in g cm^−3^). Therefore, the mass of wood (MW, in g yr^−1^) can be obtained from the dimensionally correct equation:

6 $$M{W}_{t}=BV{I}_{EW(t)}\times E{W}_{D(t)}+BV{I}_{LW(t)}\times L{W}_{D(t)}$$

*MW* series were standardized using 60 years spline functions, and the first-order autocorrelation was removed from the series. We acknowledge that tree ring standardisation and removal of autocorrelation in our tree ring series inevitably censors its lower frequency component (>50–60 years) and complicates the interpretations of teleconnection phenomena that dominate on those time scales. Ideally, to investigate those teleconnections, standardisation should be conducted based on the Regional Curve Standardisation method that was conceived precisely to overcome this problem^57^. However, it cannot be applied in our case because we do not meet the specific criteria for that method^58^ (eg. use of trees of comparable cambial ages but that lived during different periods).

### Response of *MW* to climate

Annual biomass increments (*MW*) and other density and ring width parameters at all sites were correlated to gridded land temperature and precipitation datasets over a common period: 1901–2000 (N = 100). For this purpose, we used gridded surface air temperatures (at 2 m), precipitation and sea surface temperatures from the ERA reanalysis project (1°)^59^. *MW* values averaged across EC were also compared to regional temperature and precipitation averages based on the same method. Analyses of teleconnections between MW and NAO^60^ and AMO^61^ over the last century (1901–2000) were based on standard correlation analysis and Morlet cross-spectral coherency analysis. In order to guard against the risk of spurious spectral coherencies, 90% significance levels were calculated based on the generation of 300 Monte Carlo AR(1) randomizations. Correlations were calculated to examine the mechanisms responsible for the teleconnection. Effective degrees of freedom were calculated in order to adjust *p*-values for the presence of temporal autocorrelation in the series^62^.
